# Current clinical presentations of AIDS dementia in a tropical environment: study of 26 observations in the neurology department of the University Hospital of Conakry

**DOI:** 10.1186/s40001-023-01423-w

**Published:** 2023-10-28

**Authors:** Mohamed Lamine Touré, Foksouna Sakadi, Mamady Mory Keita, Guelngar Carlos Othon, Souleymane M’bara Diallo, Thierno Hamidou Baldé, Francois Dago Kassa, Bademba Diallo, Mandandi Hinima, Mariama Boubacar Diallo, Sanny Yaya Aminou, Namory Camara, Juste Milman Kadji, Mahadi Konaté, Fode Abass Cissé, Amara Cissé

**Affiliations:** 1 Department of Neurology, CHU Ignace Deen, University Hospital of Conakry, Conakry, Guinea; 2 Neurology Department, Reference Hospital, N’Djamena, Chad; 3 Department of Radiology, University Hospital of Conakry, Conakry, Guinea; 4 Neurology Department, National Hospital, Niamey, Niger; 5 Department of Psychiatry, University Hospital of Conakry, Conakry, Guinea

**Keywords:** Dementia, HIV1 and 2, Conakry (Guinea)

## Abstract

**Introduction:**

In sub-Saharan Africa (SSA), the clinical and progressive diagnostic certainty of AIDS dementia is difficult to establish due to under-medicalization and delays in consultation and especially the diversity of etiologies of demented states.

**Material and methods:**

We carried out a retrospective study of 196 patients hospitalized for dementia syndrome between 2016 and 2021 in the neurology department of the University Hospital of Conakry.

The criteria labeled in this study are those retained by the DSM-IV and the classification of the American Academy of Neurology (AAN) developed in accordance with the WHO.

**Results:**

HIV etiology was identified in patients aged 44–67 years (17 women and 19 men). The clinical picture was dominated by severe cognitive disorders, slowed ideation, memory disorders and reduced motor skills associated with personality changes. Neurological examination revealed dysphoric disorders in most patients, sphincter abnormalities in 13 cases and labio-lingual tremor in 11 cases. Diagnosis was based on positive serological tests for HIV1 antibodies (25 cases) and HIV2 antibodies (1 case) using the Elisa and Western blot techniques, and the presence of discretely hypercellular CSF. Magnetic resonance imaging contributed to the diagnosis, showing diffuse white matter abnormalities with hyper signals on T2-weighted or FLAIR sequences.

**Conclusion:**

This study shows a non-stereotype clinical picture of AIDS dementia requiring a differential diagnosis with other infectious dementias. These results are important for the therapeutic and prognostic discussion.

## Introduction

The existence of cognitive disorders associated with HIV infection is now a well-established fact since the early publications of Janssen in 1991[1], Marder et al. in 1996 [2], Power et al. [3] and those of Fx Lescure et al. [4], Justic [5], JEVTOVIC et al. [6]. In Guinea, the prevalence of HIV is 2.8% [7], and World Health Organization data attest to a high prevalence, with one adult in 25 (3.4%) being HIV-positive, putting millions at risk of developing cognitive disorders [8].

In Guinea, the outbreak of the Ebola epidemic in 2014 [9, 10], followed recently by that of COVID-19, has led to a decline in HIV and syphilis screening, due to the dysfunction of the medical system, which is responsible for the under-reporting of sexually transmitted diseases.

Thus, in Guinea, alongside the classic neurological and psychiatric forms described with a high prevalence in sub-Saharan Africa: HIV encephalitis, toxoplasmosis, cerebrovascular accidents, progressive multifocal leukoencephalopathy [7, 8], we are witnessing the emergence of forms of progressive cognitive decline due to lax screening and prevention caused by the emergence of other epidemics. These sometimes-isolated cognitive impairment syndromes often pose a difficult etiological and clinical diagnostic problem, especially in tropical environments [10], and their link with HIV infection does not seem obvious.

The introduction of magnetic resonance imaging, improved biological exploratory techniques and the use of clinical evaluation tests for dementia in sub-Saharan Africa have enabled a better approach to the etiology of cognitive disorders of various causes, including HIV-associated dementia complex. We report 26 suspected cases of HIV-associated dementia in order to evaluate this pathology from a clinical and evolutionary point of view. The interest of this work lies in the fact that these observations illustrate AIDS dementia and the diagnostic difficulty it entails with other subcortical dementias: infectious dementias in tropical environments.

## Material and methods

Over the period from 2016 to 2021, we identified 26 cases of AIDS dementia hospitalized in the neurology and psychiatry departments of the CONAKRY university hospital, the only centers in the country for the management of nervous system diseases, from 196 patients suffering from dementia syndrome. Seventeen (17) patients came from the psychiatry department and 9 from neurology, but given the stigmatization of mental illness in Africa, all 26 patients agreed to be hospitalized in the neurology department under the supervision of psychiatrists and neurologists.

The inclusion criteria were as follows:

Patients presenting with a dementia syndrome based on DSM-V criteria (DSM-IV diagnostic and general criteria for dementia) and the American Academy of Neurology (AAN) classification developed in conjunction with WHO.

The AAN definition criteria for AIDS dementia are those of Janssen et al. [1], based on two stages: probable deficit (the patient must present each of the following points 1,2,3,4) and possible deficit with one of the following bridges (1,2).

Points 1,2,3,4 are defined in this nomenclature.

The Price and Worley criteria define the stages of AIDS dementia syndrome as stages 0–4 [13]:

- Serology by testing for anti-HIV 1 and 2 antibodies using two Elisa techniques and confirmation by Western blot [2] HIV1 25 cases and HIV2 1 case.
- No clinical, biological or radiological evidence of another etiology responsible for dementia.
- Viral studies were performed on the automated system, including PCR tests for HSV1/2, VZV, EBV, C MV and enterovirus.
- VDRL-TPHA, Hepatitis B and C serologies.

All patients underwent a series of laboratory tests (Auto Hematology Analyser 5, BK-6310, Germany), CBC, ESR, fasting blood glucose, CRP, 24-h proteinuria, electrolytes and renal function, calcium and magnesium phosphate SGOT, SGPT.

Analysis of cerebrospinal fluid (CSF) by lumbar puncture in all patients enabled cytological and biochemical evaluation using the Compact Mini VIDAS Automated Immunoassay System, Biomerieux, France, CSF protein, CSF glucose and chloride.

All patients underwent several neurological and psychiatric evaluations and, depending on the semiological presentation, otorhinolaryngological (Laryngoscope FNL-10 RP3, Pentax France), audiogram (Audiographe AD629b, interacoustique, France) and ophthalmological (ophthalmoscope IECLR6, 3000, HEINE MINI, Germany) examinations with fundus and visual acuity if possible, and a chest X-ray and electrocardiogram.

Neuroradiological MRI examinations were carried out in all 26 patients, with T1 and T2 sequences in the axial and frontal planes.

During hospitalization, two electroencephalographic tracings were performed in all patients using an electroencephalograph (EEG Nihon, Neurofax, Japan) and the tracings were classified into three (3) types:

### Type I:

- EEG with predominance of alpha rhythms of parieto-occipital topography whose amplitude is greater than 40 μvolts without pathological rhythms.
- EEG with predominance of alpha rhythms of small amplitudes up to 25 μvolts with a tendency to flattening.

### Type II:

- EEG without dominance proper with the existence of irregular alpha rhythms without the presence of pathological waves.
- EEG with theta rhythms of 4 to 6 cycles/s, especially temporo-parietal topography of low amplitude of 30 to 40 μvolts, isolated or sometimes grouped in the form of paroxysmal bursts.

### Type III:

- EEG with theta and delta rhythm showing abnormal figures.
- EEG with slowing of alpha rhythms associated with bursts of theta and delta waves.

Therapeutically, we adopted the classic treatment criteria with the following criteria for symptomatic patients: LT CD4 < 350/mm^3^; LT CD4 < 500/mm^3^ with rapid decline or HIV RNA > 30,000 copies/ml.

Treatment was based on Atriplat triple antiretroviral therapy (efavirenz / emtricitabine / tenofovir).

In total, all patients were classified according to Price and Worley's principle of stages of AIDS dementia syndrome, from 0 to 4 considered as the final stage. The Price and Worley scale assesses the severity of cognitive impairment and its impact on daily life. It comprises 5 stages, ordered from 0 (normal) to 4 (final stage), and a sub-stage (0.5) for patients with equivocal symptoms.

## Results

From January 2016 to December 2021, 26 patients were identified and hospitalized in the Neurology and Psychiatry departments of Conakry University Hospital. The duration of the illness was not specified due to the socio-cultural conceptions surrounding mental illness, which mean that all these patients first consult traditional medicine for several months. The mean age was 48.5 years, with extremes ranging from 44 to 67 years, and included 12 women and 14 men. In the history, risk factors for probable transmission were identified in 13 patients: high number of partners with polygamy (2 to 4) women in 9 cases, drug addiction and chronic alcoholism by local alcoholic substances in 3 cases, anal intercourse by homosexuality in 1 case. The use of condoms during sexual intercourse: vaginal, anal, urogenital was not recorded in any patient. Six (6) patients had a history of genital lesions probably related to a non-biologically identified sexually transmitted disease.

Clinical signs at the onset and during the course of the disease are summarized in Table 1. In our series as a whole, the infectious picture (fevers between 38 and 39.5 °C, broncho-pneumopathy, acute diarrhea) was recorded in 16 patients during the course of the disease.

**Table 1:** Symptoms and signs

	Percentage
– Early irritability with emotional control disorder	15 (57.9%)
– Social disinterest by refusal of assistance in official traditional ceremonies	13 (50%)
– Apathy	8 (30.7%)
– Signs of sadness with slowed ideation	8 (30.7%)
– Disgust with life with loss of interest	7 (26.9%)
– Insomnia, evening remission	11 (42.3%)
– Weight loss, loss of libido	6 (23%)
– Memory problems (working memory, episodic memory)	26 (100%)
– Dysphoric disorders (change, frequent mood, unmotivated crying)	21 (80.7%)
– Personality disorders self-blame, anxiety, feelings of guilt	22 (84.7%)
– Motor signs (tremor, pyramidal signs)	21 (80.7%)
– Tonic–clonic epileptiform seizures	2 (5.5%)

In all cases, the onset of the disease goes back several years.

However, given the low cultural level of our patients, all of whom initially spent several years in translational medicine, the onset stages reported here are those recorded during their first psychiatric consultation.

Using Price and Worley's stages of AIDS dementia syndrome (0–4), stages 0 and 0.5 (equivocal with subclinical) were not observed, as patients were seen late.

Patients were reassessed at stages 2, 3 and 4:

- Stage I: Three (3) patients. These patients were able to carry out the activities of daily living with obvious signs of motor disorders in the form of slowing or clumsiness of the extremities.
- Stage II: Nine (9) patients with moderate dementia: although they are able to perform daily activities, they are unable to manage their finances.
- Stage III: Eleven (11) patients with severe dementia and major intellectual incapacity.
- Stage IV: Three (3) patients in an almost vegetative state.

The tremors and motor signs observed in 21 (80.7%) patients are not pathognomonic of AIDS dementia, and present as discrete tremors on the index-index test and Romberg position, and are not cerebellar in character.

## Biological data

NFS, ESR, glycemia and transaminases biological data were normal in 16 patients and a mild hypochromic microcytic anemia was noted in 10 patients with an average hemoglobin level of 7.80 ± 2.2 g/dl. In the CSF, the count and the total proteins were almost unremarkable, except in 4 patients who had a slight variant proteinorachia between 0.42 and 0.82 g/l and all the patients presented with normo-glycorachia. The PCR reactions on CSF of HSV1 and 2, VZV, EBV, CMV, enterovirus, HHV6, ADH were negative as well as the PCR reactions on CSF of pneumococcus, meningococcus and Lyme, syphilis, listeria (CSF and blood) negative.

The dosages of vitamin B1 in the context of Gayet Wernické encephalopathy and of B12 were found to be normal. All patients at the time of hospitalization had a TCD 4 lymphocyte count greater than 350/mm^3^.

## Electroencephalographic data

**Fig.1:**
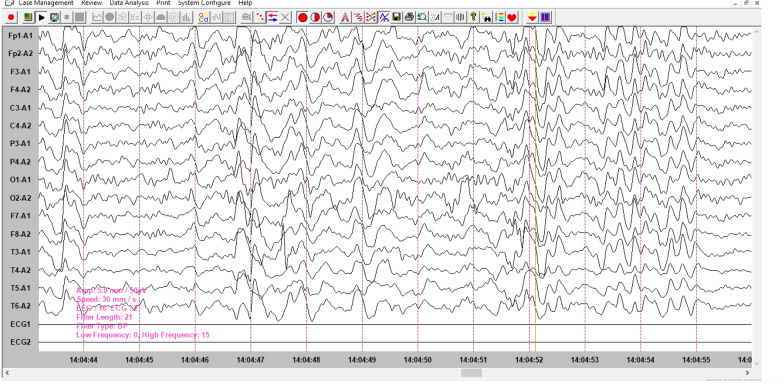
The EEG shows short generalized slow spike discharges separated by low-amplitude intervals and slow delta theta waves suggesting diffuse brain injury

**Fig.2:**
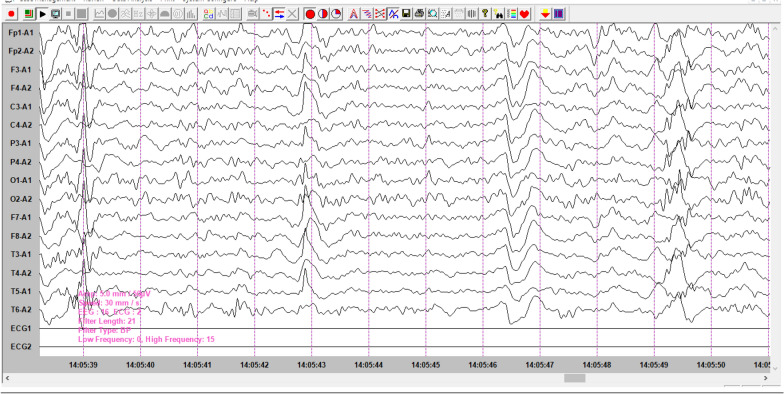
Awakening EEG tracing carried out, showing a burst of diffuse non-synchronous and periodic delta-theta slow waves at regular intervals of 6 to 7 s compatible with type III

Type I and II tracings, expression of non-specific alterations in brain bioelectrical activity were observed in 18 patients (70%) and in 8 patients (30%), the tracing reflected type III of our classification (Figs. 1, 2).

## Neuroradiological data

**Fig.3:**
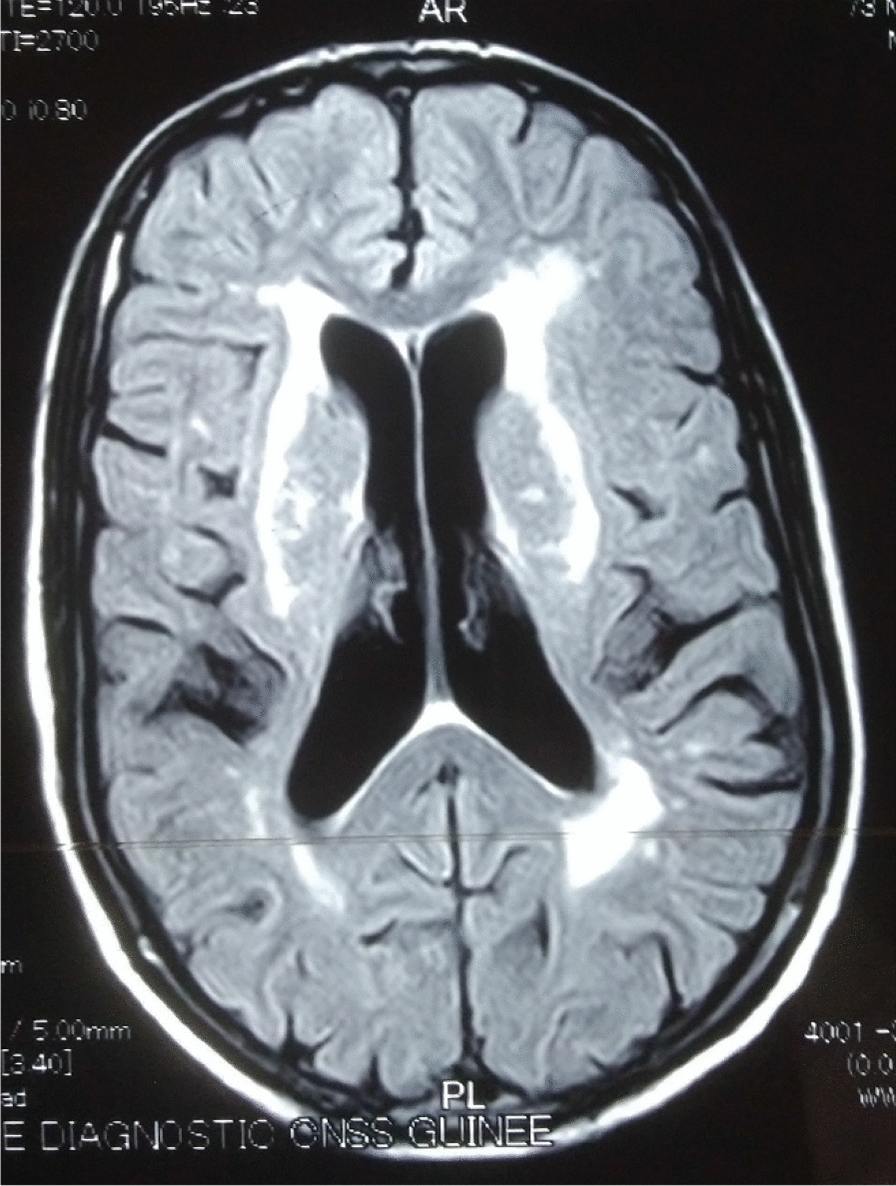
MRI cerebral: axial section FLAIR sequence abnormal white matter signal and cortical and subcortical atrophy

**Fig.4:**
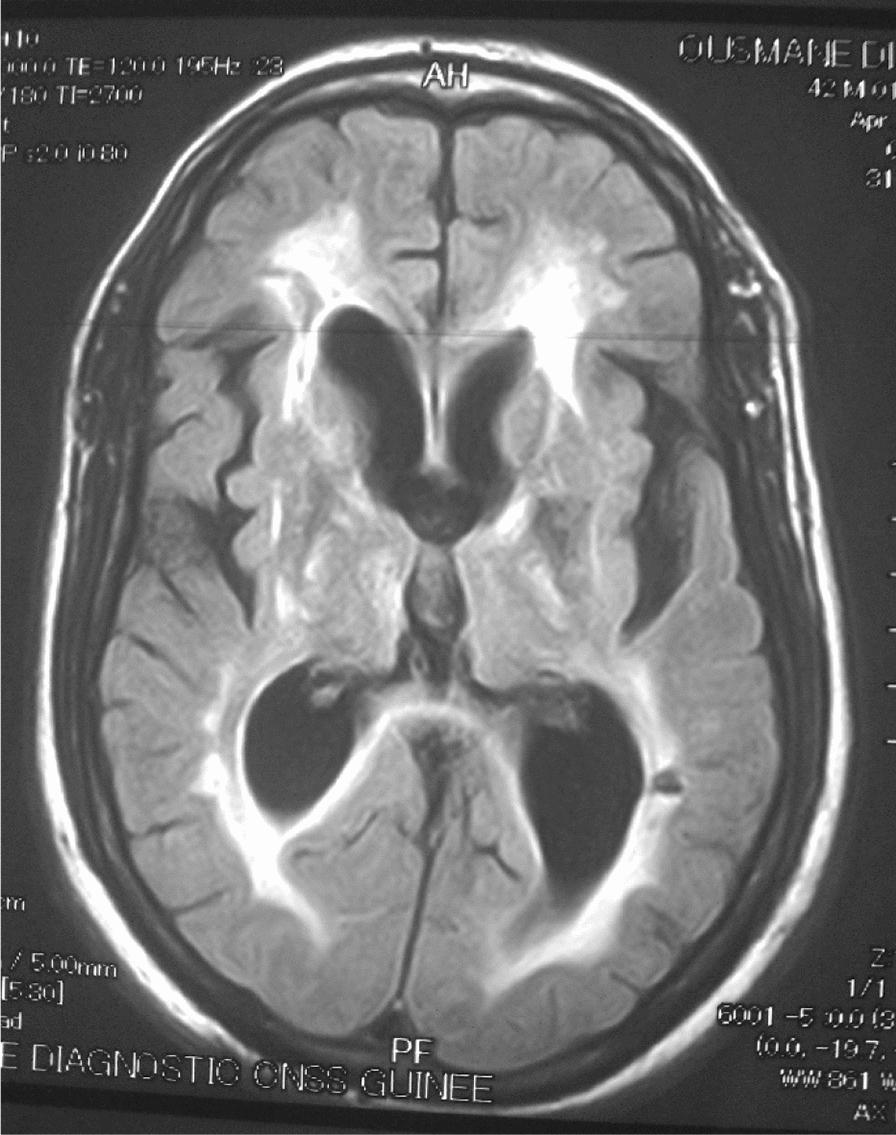
Brain MRI: axial T1 section showing areas of white matter signal abnormality

**Fig.5:**
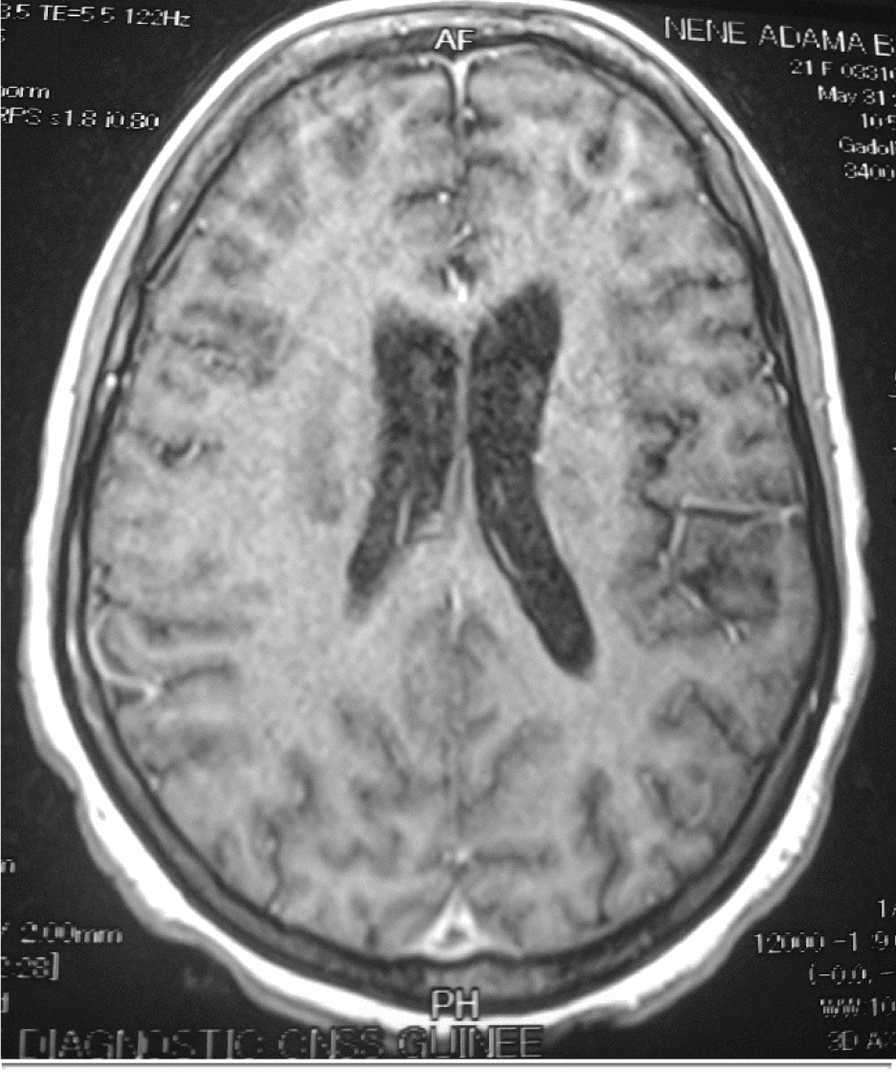
Cerebral MRI: focal abnormality in subcortical white matter, consisting of hypointense signal on T1-weighted image

**Fig.6:**
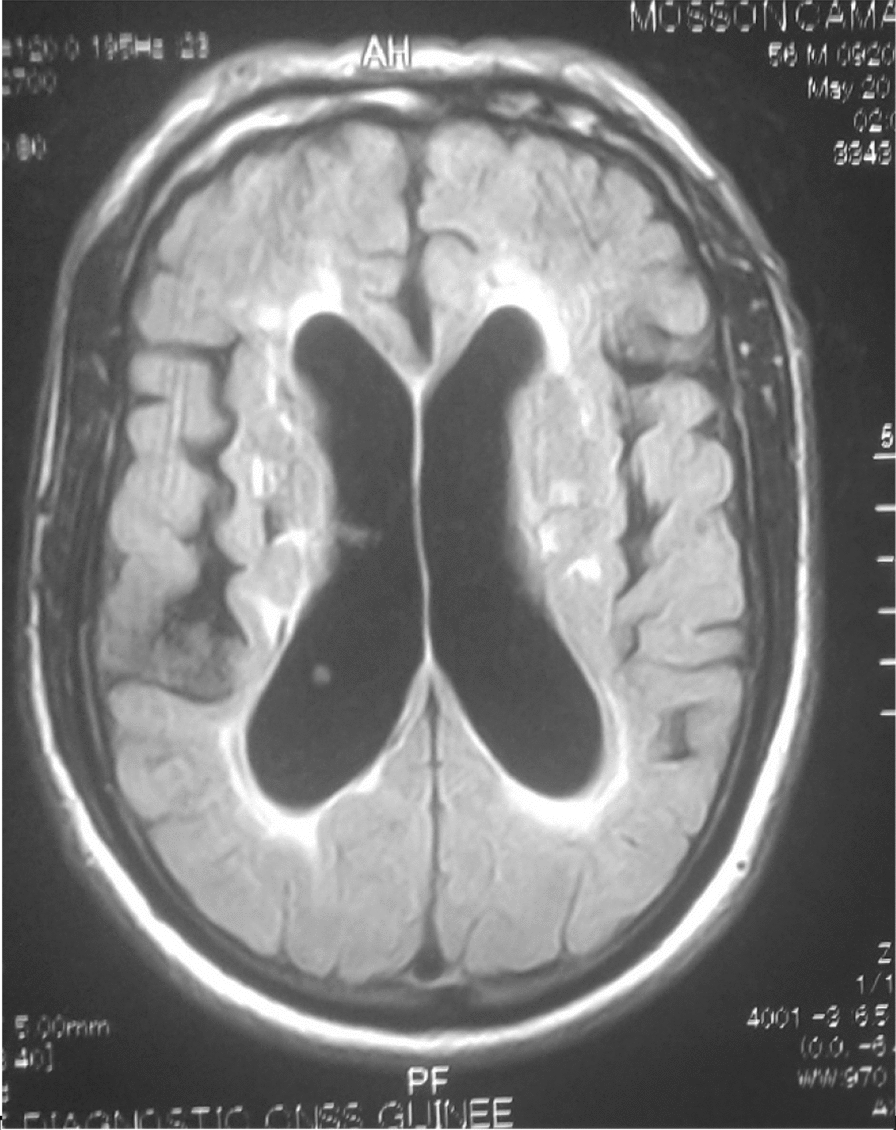
Cerebral MRI: periventricular hypersignals on the T1-weighted image

**Fig.7:**
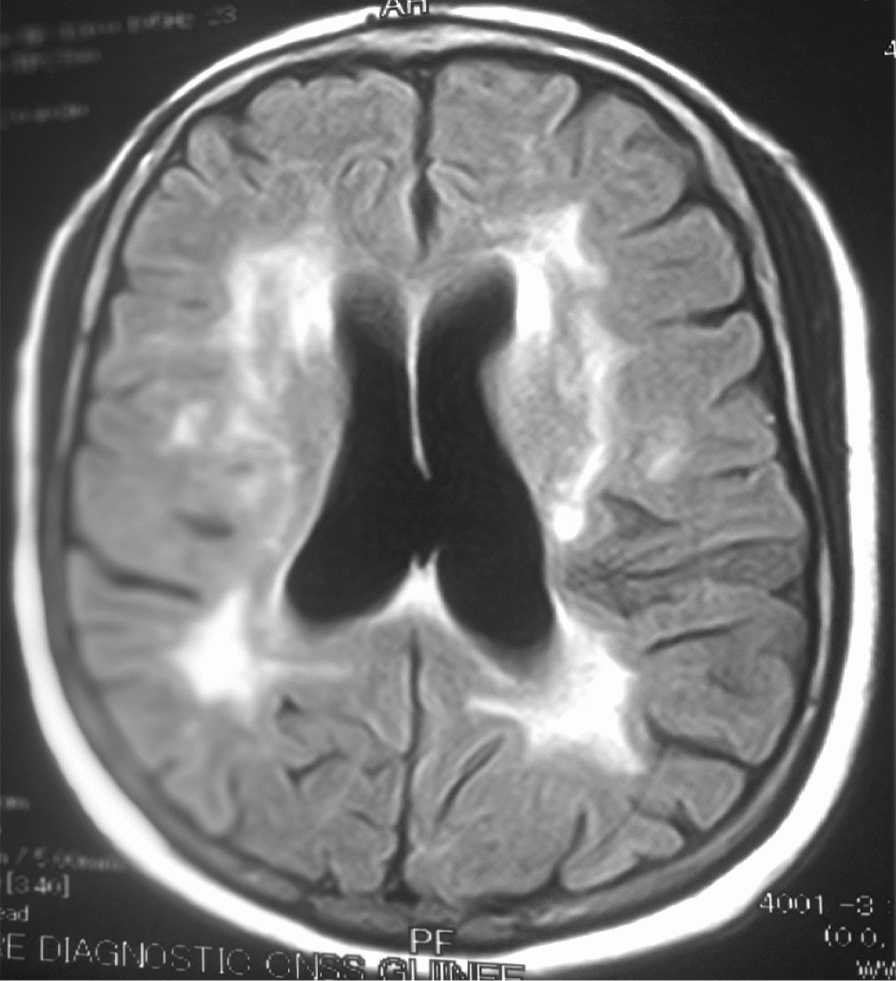
Cerebral MRI: focal abnormality in the subcortical white matter, consisting of a hypointense signal on the FLAIR-weighted image

**Fig.8:**
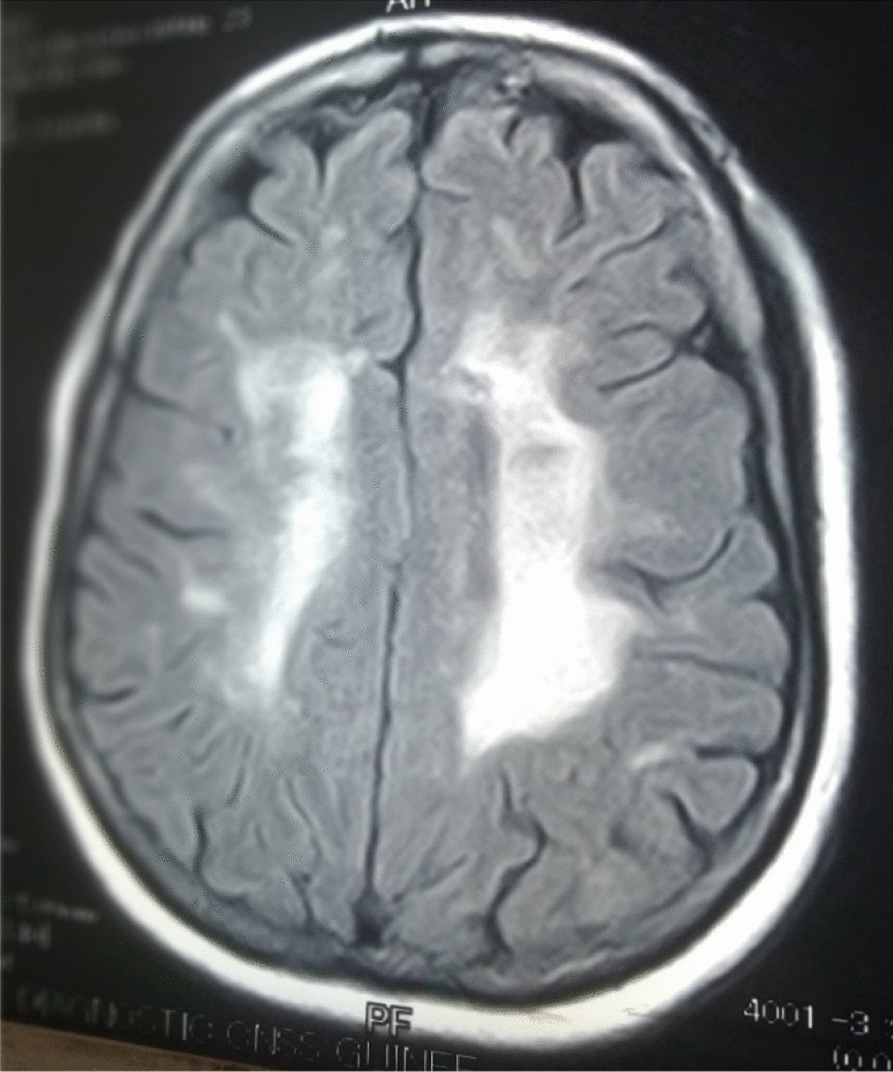
Cerebral MRI: focal abnormality in the white matter periventricular hyperintensities

**Fig.9:**
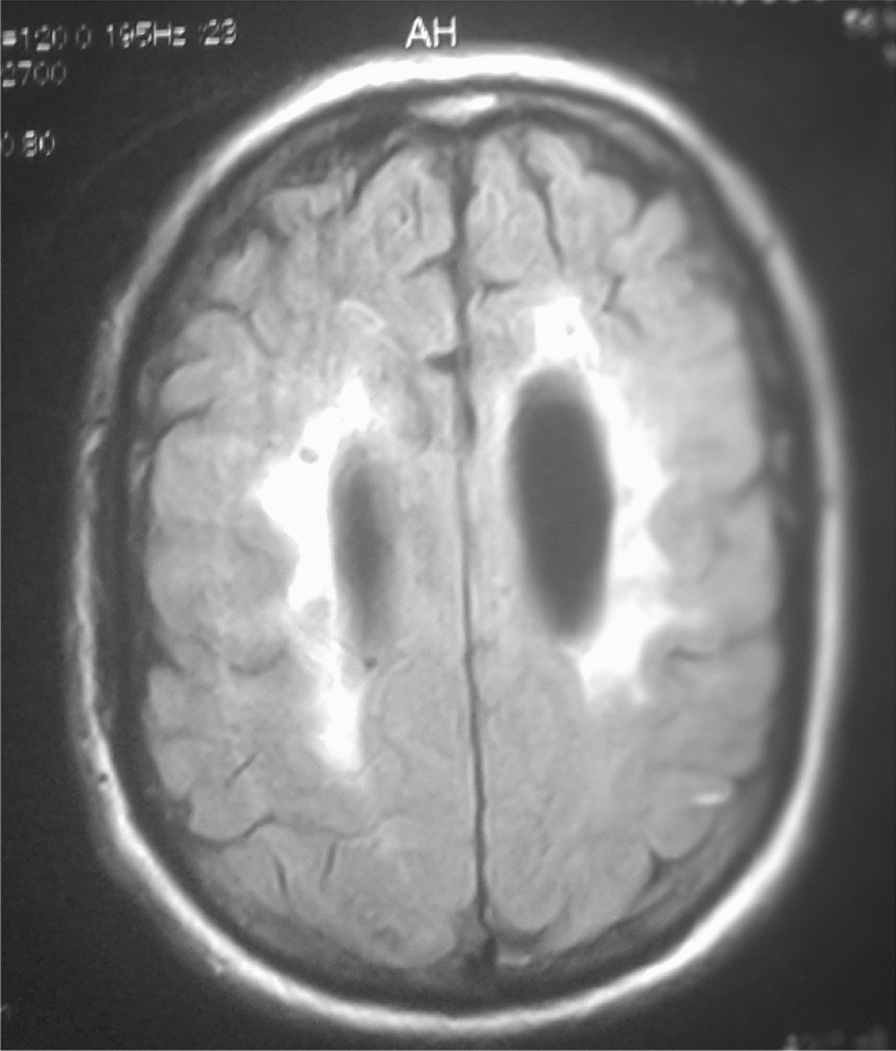
Cerebral MRI: focal abnormality in subcortical white matter, periventricular hyperintensities on T1-weighted image

**Fig.10:**
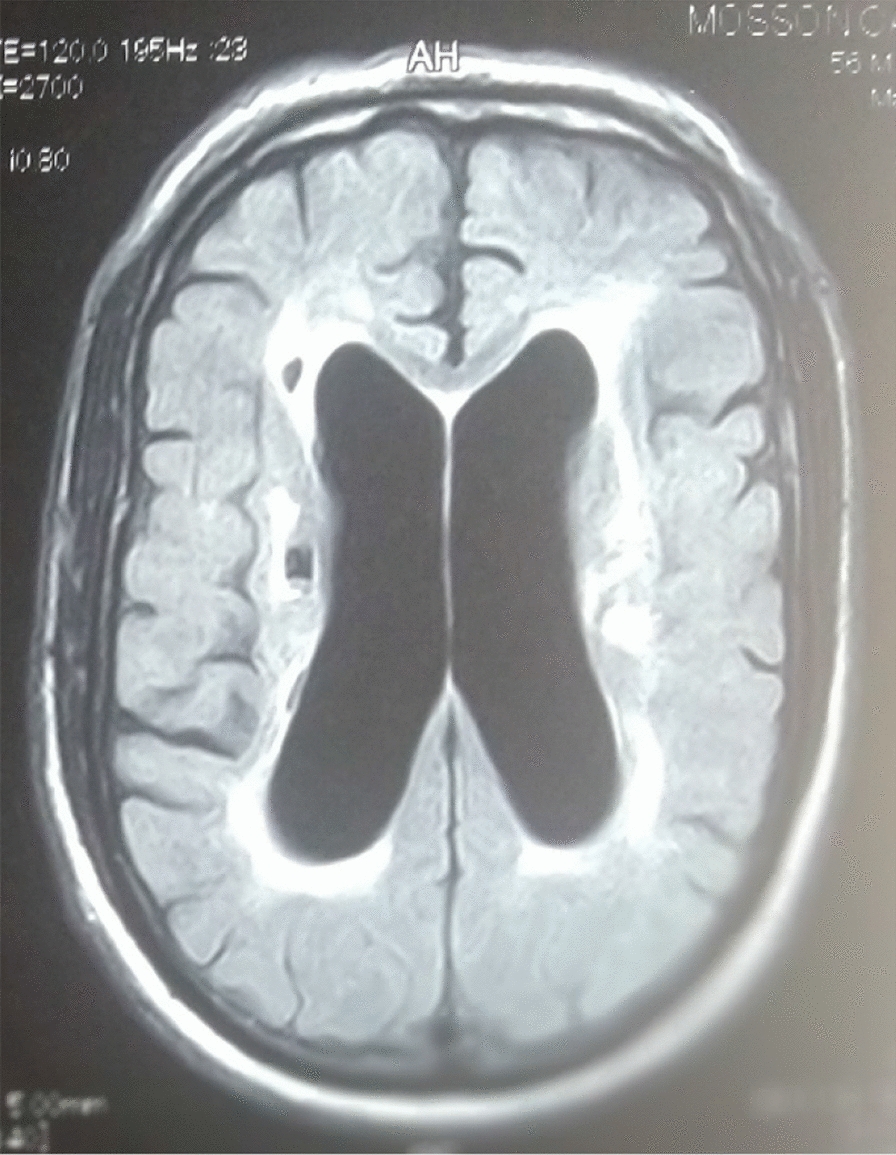
Cerebral MRI: focal abnormality in subcortical white matter, consisting of hyperintense signal on T1-weighted image

The 26 patients with a clinical picture of AIDS dementia underwent cerebral and MRI allowing differential diagnosis with vascular dementia and other subcortical dementias. In 3 cases, the MRI was normal and in 23 cases (84.5%), the MRI revealed diffuse white matter abnormalities such as hyperintensities on the T2-weighted sequences or Flair without abnormality on the sequences. weighted in T1 (Figs. 3, 4, 5, 6, 7, 8, 9, 10).

### Evolution

All patients received antiretroviral therapy (ATRIPLAT). After discharge from hospital, seven patients were lost to follow-up, and prognosis was not possible in 11 patients reviewed at 6 months and 12 months of regular follow-up; clinical symptoms regressed in patients 1 and 2 (difficulties of daily living were reduced, with improved motor initiative and no loss of dexterity); the picture remained stationary, with significant cognitive impairment and loss of autonomy in three patients, and the other three died of acute pneumonia and malaria.

## Discussion

Twenty-six observations of AIDS dementia in tropical sub-Saharan Africa were collated in a retrospective study carried out over a 5-year period in the psychiatry and neurology departments of Conakry University Hospital.

Since the initial description by Navia et al. in 1986 [11], AIDS dementia has been referred to by various authors as “AIDS dementia complex”, “subacute encephalopathy”, “HIV encephalopathy”, “HIV-associated dementia complex” and, when the picture is discrete, “HIV-associated minor cognitive disorders” [1, 12–20]. It is generally accepted that cognitive disorders occur in 30% of AIDS cases [21, 22], and some authors estimate the incidence of dementia to be around 7% per year when the CD4/mm^3^ count is below 200. Ellis et al. [23] consider that dementia can be predicted when the viral load exceeds 200 viral RNA copies/ml. In our series, asymptomatic forms were not included.

Generally speaking, the clinical pictures observed in this study do not differ fundamentally from those described in the literature, characterized essentially by memory disorders (100% of cases), psychomotor slowing, attentional difficulties, simulating Moria's neuropsychological syndrome, expressed by intellectual, thymic and behavioral disorders [24–28]. The low cultural level of our patients, and the stigmatization of mental illness and AIDS still in force, have prevented a systematic description of severe psychiatric disorders, notably obsessional manifestations, hallucinations, the self-accusation syndrome and major depressive states, although these have been described by several authors in AIDS dementia [29, 30]. This clinical symptomatology has been described by most authors [31], especially as our patients were seen at Price and Worley stages 2 and 3 [13].

In this study, amnesic disorders were aggravated by the stigmatizing socio-cultural conceptions noted in diseases with mental connotations, with the appearance of major psychiatric manifestations in some patients: anxiety, obsessive rumination, self-accusation and even suicidal ideation, an expression of major depression in these patients.

The reported memory disorders, intellectual slowing, heightened by the depressive psychiatric symptomatology observed, correspond to the known clinical and neuropsychological characteristics of this condition. Severe forms exist, and under-medicalization is a further explanation for the severity of psychiatric disorders, which are also diagnosed late.

Cognitive disorders are also multifactorial, hence the importance of an exhaustive blood test when managing them, in particular to look for another cause, such as infection with toxic substances, as demonstrated by the use of locally produced alcohol by some of our patients.

Biologically, most authors note that lumbar puncture does not reveal CSF abnormalities, although Moulignier et al. [12] and other series confirm the existence of isolated lymphocytic meningitis [32–37]. The same studies confirm that viral load is not currently a contributing factor in assessing the quantity and severity of dementia [5]. Imaging findings in AIDS dementia have been reported by several authors [38–40], all emphasizing diffuse abnormalities of the substance in the form of hypersignals on T2-weighted sequences or FLAIR under abnormalities on T1-weighted sequences (Figs. 3, 4, 5, 6). Our study also shows the importance of MRI in the diagnosis of AIDS dementia. It can also be used to rule out other differential diagnoses, a fortiori or in the event of an advanced encephalic picture discovered late.

EEG data are dominated by types 1 and 2, expressing non-specific cortico-subcortical distress, and in a third of cases, unsystematized slow-wave puffs may be encountered, although paroxysmal activity may be present in 2 cases.

## Conclusion

This study recounts the clinical and psychiatric characteristics of 26 cases of AIDS dementia, manifested by memory disorders, psychomotor slowing and psychiatric disorders.

This study confirms the presence and persistence of HIV in Guinea, with inadequate treatment, thus calling for its improvement.

## Data Availability

Not applicable.
